# Profilometric evaluation of root surface roughness using pumice and zirconium silicate polishing agents

**DOI:** 10.6026/973206300210268

**Published:** 2025-02-28

**Authors:** Devadharshini Chandrasekar, C. Burnice Nalina Kumari, Nikita Ravi, Jaideep Mahendra, Vijayalakshmi Rajaram

**Affiliations:** 1 Department of Periodontics, Meenakshi Ammal Dental College and Hospital, Chennai, Tamil Nadu, India

**Keywords:** Periodontitis, tooth polishing, profilometry, root surface roughness, pumice polishing powder, zirconium silicate prophylaxis paste

## Abstract

Periodontitis is a chronic inflammatory disease that leads to tooth loss with dental plaque and calculus as key risk factors. Periodontal prophylaxis including polishing plays a crucial role in reducing plaque deposition and maintaining periodontal health. Therefore, it is of interest to compare the root surface roughness produced by pumice polishing powder and zirconium silicate prophylaxis paste using a profilometric analysis on the extracted teeth. The results show that zirconium silicate paste produced a smooth root surface compared to pumice powder which exhibited a rough texture. Thus, zirconium silicate paste is preferable for preserving root surface integrity during periodontal prophylaxis.

## Background:

One of the most widespread diseases affecting the oral cavity is periodontitis. The condition must be treated quickly since it not only causes tooth loss but also has an impact on the patient's general health [1]. The interactions between the colonised layer of microbial dental plaque and the non-specific and specific host responses on the gingival side lead to periodontitis [2]. Treatment of periodontal disease can be achieved by maintaining control over the oral biofilm and eliminating bacterial plaque. The cornerstone of periodontal therapy is scaling and root planing (SRP), which plays a crucial role in maintaining periodontal health and prevents the recurrence of the disease [3]. Another crucial factor in periodontal treatment is the instrumentation-induced roughness of the remaining root surface [4]. Tooth polishing is defined as "the removal of plaque, calculus and stains from the exposed and unexposed surfaces of the teeth by scaling and polishing as a preventive measure for the control of local irritational factors" [5]. The main goal of polishing is to create the smoothest surface possible by eliminating stains and bacterial buildup. Polishing is the last phase of periodontal therapy following SRP. Presently, numerous polishing techniques are employed, that includes the air powder system, the revolving rubber cup, the nylon bristle brush, polishing paste and pumice [6]. It should be noted that each of these polishing techniques has unique benefits and drawbacks regarding the roughness of the enamel and root surface. Therefore, it is of interest to compare the root surface roughness produced by pumice polishing powder and zirconium silicate prophylaxis paste using a profilometric analysis on the extracted teeth.

## Materials and Methods:

The present study was carried out on 30 human extracted single rooted teeth comprising of incisors, canines and premolars which were lost due to periodontal and orthodontic reasons.

## Sample preparations and group divisions:

After the tooth was extracted, it was washed under running water for 1 min and then it was transferred and maintained in 10% formalin. All the extracted teeth were scaled by a calibrated operator using the ultrasonic device to remove residual calculus and tissue tags. On visual inspection, the surface appeared smooth and clean which denoted satisfactory scaling. Then the specimens were completely planed with 1-2, 3-4 Gracey curettes to remove the altered cementum and the specimens were then preserved in normal saline until further study.

The teeth were randomly divided into three groups so that 10 teeth were present in each group.

[1] Group A - Control Group (No polishing done)

[2] Group B - Polishing done with Pumice Powder

[3] Group C - Polishing done with Zirconium silicate prophylaxis paste

**Figure 2:**
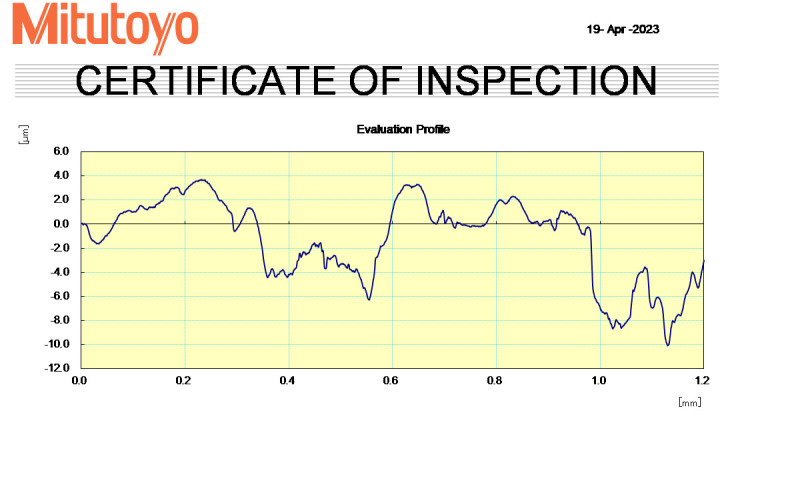
Graph obtained by running the stylus on the tooth surface

Each group was put through an analysis by applying a stylus profilometer to assess the surface topography. These samples were positioned on the profilometer in the manner shown (Figure 1 - see PDF). The apparatus was calibrated and optimised to allow the stylus to go up to 4 mm apicocoronally. The stylus was moved concurrently and a representative graph was produced (Figure 2).

## Results:

Throughout the study, the parameters assessed were as follows:

[1] Ra - Average of roughness profile, is the arithmetic average of the absolute values of the profile heights over the evaluation length

[2] Rq - Root mean square Roughness, is the root mean square average of the profile heights over the evaluation length

[3] Rz - Average Maximum Height of the Profile, is the average of the successive values of Maximum heights within the sampling length calculated over the evaluation length.

**Figure 3:**
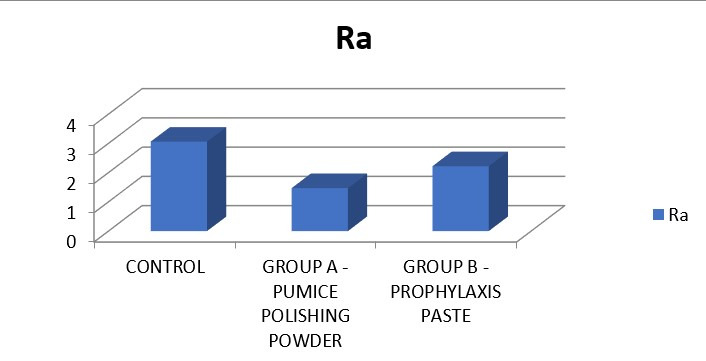
In Ra highest mean was seen in control followed by group B and group A. The comparison was found to be statistically significant.

**Figure 4:**
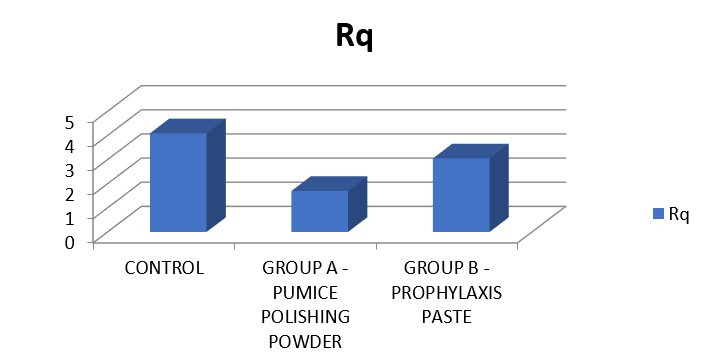
In Rq highest mean was seen in control followed by group B and group A. The comparison was found to be statistically significant.

**Figure 5:**
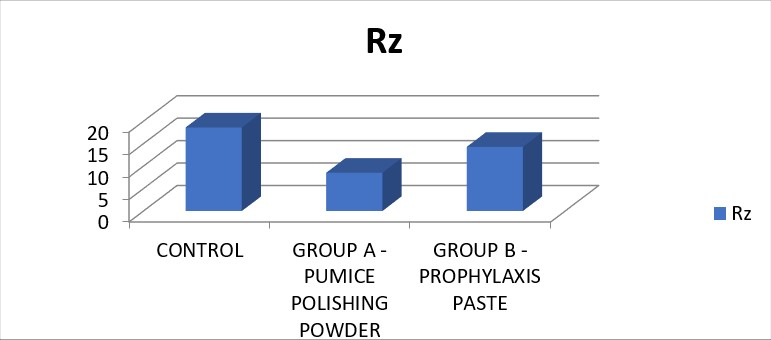
In Rz highest mean was seen in control followed by group B and group A. The comparison was found to be statistically significant.

In Ra, highest mean was seen in control followed by group C and group B (Figure 3). The comparison was found to statistically significant. In Rq, highest mean was seen in control followed by group C and group B (Figure 4). The comparison was found to be statistically significant. In Rz, highest mean was seen in control followed by group C and group B (Figure 5). The comparison was found to be statistically significant (Table 1). The post hoc pairwise comparisons were calculated and were found to be statistically significant (Table 2).

On analysis of the Ra, the mean surface roughness in Group A (No polishing done) was 3.06. In Group B (Pumice powder), the mean surface roughness was 1.47. In Group C (Prophylaxis paste) exhibited a mean roughness of 2.22. On analysis of the Rq, the mean root square roughness in Group A (No polishing done) was 4.09. In Group B (Pumice powder), the mean surface roughness was 1.70. In Group C (Prophylaxis paste) exhibited a mean roughness of 3.05. On analysis of the Rz, average maximum height of the profile in Group A (No polishing) was 18.62. In Group B (Pumice powder), the mean surface roughness was 8.53. In Group C (Prophylaxis paste) exhibited a mean roughness of 14.30. The differences between the three groups were statistically significant (p< 0.01). Intergroup comparisons, established by Post Hoc Tests pairwise comparison the mean difference is significant at the 0.05 level. Based on Ra the mean difference between Group A with Group B and Group C was 1.591 and 0.842 respectively , the significant mean difference on comparing Group B with Group A and Group C was -1.591 and -0.749 respectively , the significant mean difference on comparing Group C with Group A and Group B was -0.842 and 0.749 respectively. With respect to Rq it was noted that, the statistically significant mean difference between, Group A and Group B was 2.390, Group B with Group A and Group C was -2.390 and -1.344 respectively and Group C with Group B was 1.344. On analysing Rz the mean difference between Group A and Group B was 10.084 and that of Group B with Group A was -10.084. The above mentioned mean differences were statistically significant.

## Discussion:

Following prophylactic procedures, the root surface's roughness can promote persistent plaque build-up, especially on the proximal areas, which will promote the emergence of gingival inflammation [7]. Plaque, biofilm and stains on the enamel and root surfaces must be removed in order to create the smoothest surface possible. The desired outcome for effective root planing should be a smooth root surface [8]. After mechanical debridement, a smooth surface is created which is designed to help in the reattachment of gingival fibrous tissues. The surface characteristics of the tissue are crucial for tissue regeneration in addition to physiological tissue repair [9]. The aim of the present *in-vitro* study was to analyse and compare the root surface roughness produced by two commercially available polishing agents *i.e.* Pumice polishing powder and Zirconium silicate prophylaxis paste. Prior to the polishing procedures, the enamel and root surface of the specimens were standardized by complete ultrasonic scaling and thorough root planning. The results of the current study demonstrate that polishing significantly reduces the roughness of the tooth surface. The present study established that Pumice powder produced less surface roughness on the root surface when compared to Zirconium silicate prophy paste. The larger particle size of the polishing paste than pumice could be attributed to these outcomes. The findings of present study were similar with the study done by Yildrim *et al.* (2021) [10].

The polishing procedures may be carried out using a wide range of materials and techniques. Studies revealed that polishing subsequently after scaling reduced bacterial deposits, eliminated plaque and refined the tooth surface [11]. Negative consequences from polishing like, tooth abrasion and dentin hypersensitivity, may still manifest [12]. The outcomes of the present study additionally implied that polishing techniques annexed with SRP procedures yielded smoother surfaces than SRP techniques alone. As a result, polishing was advised following SRP procedure [11]. To achieve the ideal smooth surface, the form of the powder particles is very important. In our study, we employed a profilometer to evaluate the roughness of the root surface. Profilometer is a device used specifically to measure surface roughness of any material [13]. Reduced surface roughness and debris on both enamel and cement surfaces were also seen in studies by Leknes, Lie and Patil *et al.* [14, 15]. Incorporating profilometers, studies by Cuesta *et al.* and Kayahan *et al.* analysed surface roughness in metallurgy [16, 17]. Implementing a profilometer provides the benefit of being economical and rapid for analysis. Profilometric analysis will not require a meticulously prepared sample. Analysis and findings can be acquired immediately. It precisely maps the surface of any material using laser guiding [13]. Size, shape and hardness of the powder have a significant impact on abrasiveness of the tooth surface [18]. Contrary to the findings of Jana *et al.* (2016), which emphasize the necessity of submicron-sized particles for achieving optimal root surface smoothness (Ra <0.2 µm), our study demonstrates that pumice, despite its larger particle size, produces a smoother root surface compared to zirconium silicate prophylaxis paste. This suggests that particle composition and mechanical properties may have a more significant impact on polishing efficacy than particle size alone, highlighting the need for further research to refine the selection of optimal polishing agents for periodontal prophylaxis [19].

A comparative profilometric evaluation of pumice polishing powder and zirconium silicate prophylaxis paste by quantifying root surface roughness with the clinical significance for selecting an optimal polishing agent to periodontal prophylaxis is documented. It highlights the role of particle size in achieving a biologically favourable root surface, reinforcing the importance of submicron particles for effective polishing. However, as an *in-vitro* study, it lacks intraoral conditions such as saliva, biofilm formation and masticatory forces, limiting its direct clinical applicability. The sample size restricts broader generalization and the study evaluates only two agents, necessitating further research on other commercially available materials. Future studies should focus on in vivo assessments of polishing agents' long-term effects on biofilm adherence and periodontal healing, incorporating advanced imaging techniques like scanning electron microscopy for a more detailed surface analysis.

## Conclusion:

Polishing is essential for refining root surfaces by eliminating irregularities caused by mechanical instrumentation. A comparative evaluation of pumice powder and zirconium silicate prophylaxis paste revealed that pumice powder resulted in significantly lower surface roughness. These findings emphasize the importance of selecting appropriate polishing agents to optimize root surface integrity and enhance periodontal prophylaxis outcomes. However, further in vivo studies are needed to assess the long-term effects of different polishing agents on periodontal healing and biofilm adherence. Additionally, advanced imaging techniques such as scanning electron microscopy (SEM) could provide deeper insights into surface modifications post-polishing.

## Figures and Tables

**Table 1:** Onaway ANOVA test: intergroup comparison of average roughness profile

		**N**	**Mean**	**Std. Deviation**	**P value**
Ra	GROUP A - NO POLISHING DONE	10	3.06	0.519	0
	GROUP B - PUMICE POLISHING POWDER	10	1.47	0.244	
	GROUP C - PROPHYLAXIS PASTE	10	2.22	0.86	
	Total	30	2.25	0.876	
Rq	GROUP A - NO POLISHING DONE	10	4.09	1.268	0
	GROUP B - PUMICE POLISHING POWDER	10	1.7	0.45	
	GROUP C - PROPHYLAXIS PASTE	10	3.05	1.511	
	Total	30	2.95	1.503	
Rz	GROUP A - NO POLISHING DONE	10	18.6	6.812	0.004
	GROUP B - PUMICE POLISHING POWDER	10	8.53	2.28	
	GROUP C - PROPHYLAXIS PASTE	10	14.3	7.968	
	Total	30	13.8	7.305	

**Table 2:** Post hoc tests pairwise comparison: correlative multiple comparisons between control and test groups

**Multiple Comparisons**							
**Tukey HSD**							
Dependent Variable	(I) GROUP	(J) GROUP	Mean Difference (I-J)	Std. Error	Sig.	95% Confidence Interval	
						**Lower Bound**	**Upper Bound**
Ra	GROUP A - NO POLISHING DONE	GROUP B - PUMICE POLISHING POWDER	1.591*	0.267	0	0.93	2.25
		GROUP C - PROPHYLAXIS PASTE	.842*	0.267	0.01	0.18	1.5
	GROUP B - PUMICE POLISHING POWDER	GROUP A - NO POLISHING DONE	-1.591*	0.267	0	-2.25	-0.93
		GROUP C - PROPHYLAXIS PASTE	-.749*	0.267	0.02	-1.41	-0.09
	GROUP C - PROPHYLAXIS PASTE	GROUP A - NO POLISHING DONE	-.842*	0.267	0.01	-1.5	-0.18
		GROUP B - PUMICE POLISHING POWDER	.749*	0.267	0.02	0.09	1.41
Rq	GROUP A - NO POLISHING DONE	GROUP B - PUMICE POLISHING POWDER	2.390*	0.522	0	1.09	3.69
		GROUP C - PROPHYLAXIS PASTE	1.046	0.522	0.13	-0.25	2.34
	GROUP B - PUMICE POLISHING POWDER	GROUP A - NO POLISHING DONE	-2.390*	0.522	0	-3.69	-1.09
		GROUP C - PROPHYLAXIS PASTE	-1.344*	0.522	0.04	-2.64	-0.05
	GROUP C - PROPHYLAXIS PASTE	GROUP A - NO POLISHING DONE	-1.046	0.522	0.13	-2.34	0.25
		GROUP B- PUMICE POLISHING POWDER	1.344*	0.522	0.04	0.05	2.64
Rz	GROUP A - NO POLISHING DONE	GROUP B - PUMICE POLISHING POWDER	10.084*	2.77	0	3.22	16.95
		GROUP C - PROPHYLAXIS PASTE	4.32	2.77	0.28	-2.55	11.19
	GROUP B - PUMICE POLISHING POWDER	GROUP A - NO POLISHING DONE	-10.084*	2.77	0	-16.95	-3.22
		GROUP A - PROPHYLAXIS PASTE	-5.764	2.77	0.11	-12.63	1.1
	GROUP C - PROPHYLAXIS PASTE	GROUP A - NO POLISHING DONE	-4.32	2.77	0.28	-11.19	2.55
		GROUP B - PUMICE POLISHING POWDER	5.764	2.77	0.11	-1.1	12.63
*. The mean difference is significant at the 0.05 level
